# Marjolin's ulcers at a university teaching hospital in Northwestern Tanzania: a retrospective review of 56 cases

**DOI:** 10.1186/1477-7819-10-38

**Published:** 2012-02-15

**Authors:** Phillipo L Chalya, Joseph B Mabula1, Peter Rambau, Mabula D Mchembe, Kahima J Kahima, Alphonce B Chandika, Geofrey Giiti, Nestory Masalu, Robert Ssentongo, Japhet M Gilyoma

**Affiliations:** 1 Department of Surgery, Catholic University of Health and Allied Sciences-Bugando, Mwanza, Tanzania; 2 Department of Pathology, Catholic University of Health and Allied Sciences-Bugando, Mwanza, Tanzania; 3 Department of Oncology, Catholic University of Health and Allied Sciences-Bugando, Mwanza, Tanzania; 4 Department of Surgery, Muhimbili University of Health and Allied Sciences, Dar Es Salaam, Tanzania; 5 Department of Plastic and Reconstructive Surgery, Mulago Hospital Complex, Kampala, Uganda

**Keywords:** Marjolin's ulcers, clinicopathological pattern, treatment outcome, Tanzania

## Abstract

**Background:**

Marjolin's ulcer is a rare but highly aggressive squamous cell cancer that is most often associated with chronic burn wounds. Although many individual case reports exist, no comprehensive evaluation of Marjolin's ulcer patients has been conducted in our setting. This study was conducted to describe the clinicopathological presentation and treatment outcome of this condition in our local setting and to identify predictors of outcome.

**Methods:**

This was a retrospective study of histologically confirmed cases of Marjolin's ulcer seen at Bugando Medical Centre over a period of 10-years between January 2001 and December 2010. Data were retrieved from patients' files and analyzed using SPSS computer software version 15.0

**Results:**

A total of 56 patients were studied. Male to female ratio was 2.1:1. Burn scars (89.3%) were the most common causative lesions of Marjolin's ulcer. The mean latent period between original injury and diagnosis of Marjolin's ulcer was 11.34 ± 6.14 years. Only 12.0% of the reported cases were grafted at the time of injury (*P *< 0.00). Most patients (48.2%) presented between one and five years of onset of illness. The lower limb (42.9%) was the most frequent site for Marjolin's ulcers. The median tumor size at presentation was 8 cm and the vast majority of patients (85.7%) presented with large tumors of ≥ 5 cm in diameter. Lymph node metastasis at the time of diagnosis was recorded in 32.1% of cases and distant metastasis accounted for 26.9% of cases. Squamous cell carcinoma (91.1%) was the most common histopathological type. Wide local excision was the most common surgical procedure performed in 80.8% of cases. Post-operative complication rate was 32.1% of which surgical site infection was the most common complication in 38.9% of patients. Local recurrence was noted in 33.3% of cases who were treated surgically. The mean length of hospital stay for in-patients was 7.9 ± 2.3 days. Mortality rate was 7.1%. According to multivariate logistic regression analysis, stage and grade of the tumor and presence of local recurrence were the main predictors of death (P < 0.001).

**Conclusion:**

Marjolin's ulcers are not rare in our environment and commonly occur in burn scars that were not skin grafted and were left to heal secondarily. A high index of suspicion is required in the management of chronic non-healing ulcers and all suspected lesions should be biopsed. Early recognition and aggressive treatment of Marjolin's ulcers and close follow-up are urgently needed to improve outcomes in our environment.

## Background

Marjolin's ulcer is a rare and often aggressive cutaneous malignancy that arises in previously traumatized or chronically inflamed skin, particularly after burns [1]. Marjolin's ulcers have a 1% to 2% incidence in all burn scars but can also develop from previously traumatized and scarred tissue of other etiologies such as chronic sinuses of osteomyelitis, post-traumatic wounds, pressure sores and chronic fistulae and have been reported to arise in the genitalia as a complication of Fournier's gangrene [2]. The term "Marjolin's ulcer" was named after French surgeon, Jean Nicolas Marjolin, who first described the condition in 1828 [1-3]. But it was Dupuytren who noted it was a malignancy [4]. In 1923, DaCosta first coined the expression "Marjolin's ulcer" to describe malignant tumors forming over burn injuries [5].

The exact mechanism of malignant transformation of Marjolin's ulcer remains unclear and controversial [6-8]. Several theories including the toxin, chronic irritation, traumatic epithelial elements implantation, heredity, immunologic privileged site, co-carcinogen, ultraviolet rays, initiation and promotion and environmental and genetic interaction theories have been reported to explain the malignant transformation [9].

The latency period from the time of injury to the onset of malignant transformation averages 36 years [10]. However, early arising Marjolin's ulcers have been described in the literature [8,11]. Studies from western countries have shown that the average age at diagnosis is in the fifth decade of life with a range of 18-84 years, and men are three times more frequently affected than women [11]. In Sub-Saharan Africa, Marjolin's ulcer appears to be affecting younger patients and the transition time is getting shorter over the years [12].

Most lesions of Marjolin's ulcer occur on the extremities (60%), with ulcers on the head and face occurring less frequently (30%) and the lowest frequency (10%) on the trunk [4]. Marjolin's ulcers in unusual sites such as the genitalia as a complication of Fournier's gangrene [13] and breast skin developing in a postburn scar [14] have also been reported.

Marjolin's ulcers are very aggressive tumors that necessitate a well thought out treatment plan to optimize care and assure patient survival [14]. Early diagnosis and prompt surgical intervention is mandatory in Marjolin's ulcers as they may invade vital structures. However, in developing countries like Tanzania, most patients with these lesions are treated as chronic ulcers or infections and present late to hospitals, leading to a delayed diagnosis and resulting in the need for more extensive surgery and an increased risk of metastasis.

Marjolin's ulcers presents histopathologically as squamous cell carcinoma in 75% to 96% of cases [15]. Other neoplasms, such as basal cell carcinomas, melanoma, osteogenic sarcoma, fibrosarcoma, and liposarcoma, have also been reported [8,16].

In spite of being a surface malignancy and more amenable not only to early detection, but also to a potential cure, the outcome of treatment in most developing countries including Tanzania has been poor because the majority of these patients present late to the hospital with advanced stage. This is partly due to paucity of local data regarding this condition and lack of community awareness on the importance of early reporting to hospital for early diagnosis and treatment. This study was conducted to evaluate the clinicopathological presentation and treatment outcome of this condition in our local setting and to identify predictors of outcome.

## Methods

### Study design and setting

This was a retrospective study of histologically confirmed cases of Marjolin's ulcer seen at the department of Surgery of Bugando Medical Centre (BMC) over a period of 10-years between January 2001 and December 2010. BMC is a consultant, tertiary care and teaching hospital for the Catholic University of Health and allied Sciences-Bugando (CUHAS-Bugando) and has a bed capacity of 1000.

### Study population

The subjects of this study included all patients who presented to BMC with histologically confirmed Marjolin's ulcers during the period studied. Patients with incomplete data were excluded from the study. The details of patients were retrieved from patients' files kept in the Medical record department, the surgical wards, operating theatre and histopathology laboratory. Information retrieved included demographic data, causative previous lesions, latency period, duration of illness, anatomical site, radiological investigations, histological types, treatment modalities, management complications, length of hospital stay and mortality.

### Statistical analysis

Data collected were analyzed using SPSS computer software version 15.0 (SPSS, Inc, Chicago, IL). Data was summarized in form of proportions and frequency tables for categorical variables. Continuous variables were summarized using mean, median, mode and standard deviation. Chi -square (χ^2^-test) was used to test for significance of associations between the predictor and outcome variables in the categorical variables. Student t-test was used to test for significance of associations between the predictor and outcome variables in the continuous variables. Significance was defined as a p-value of < 0.05. Multivariate logistic regression analysis was used to determine predictor variables that are associated with outcome.

### Ethical consideration

Ethical approval to conduct the study was sought from the CUHAS-Bugando/BMC joint institutional ethic review committee before the commencement of the study.

## Results

### Demographic data

During the period under study, a total of 56 patients were studied. Males were 38 (67.9%) and females were 18 (32.1%) with a male to female ratio of 2.1:1. Their mean and median ages were 38.2 ± 24.2 years and 37 years respectively (range 12-78 years). The modal age group was 31-40 years. The majority of patients, 39 (69.6%) were younger than 40 years. The vast majority of patients, 49 (87.5%) came from the rural areas located a considerable distance from Mwanza City and most of them, 51 (91.1%) had either primary or no formal education.

### Causative previous lesions

Burn scars were the most common causative lesions of Marjolin's ulcer, representing 89.3% of cases. The mean latent period between original injury and development of Marjolin's ulcer was 11.34 ± 6.14 years (range from five months to 48 years). Skin grafting of the previous causative lesions was reported in only six (10.7%) patients and the rest of the lesions healed by either delayed primary or secondary intention.

### Clinical presentation

The duration of illness (the period from development of Marjolin's ulcer and presentation to health facility) ranged from 1 month to 8 years with a median of 4 years and the majority of patients (48.2%) presented between one and five years of onset of illness. The reasons for late presentation are shown in Table 1. Forty-six (82.1%) patients had undergone some form of intervention in peripheral dispensaries and hospitals before been referred, with dressing and inadequate surgical resection being the most common intervention.

**Table 1:** Clinical presentation

Variables	Responses	Frequency	Percentage
**Previous lesions**	Burn scars	50	89.3
	Penile human bite scar	1	1.8
	Pressure sores	1	1.8
	Chronic non healing ulcer	1	1.8
	Chronic osteomyelitis	1	1.8
	Diabetic foot ulcer	1	1.8
	Fournier's gangrene	1	1.8
**Duration of illness (years)**	< 1	13	23.2
	1-5	27	48.2
	> 5	16	28.6
**Reason for late presentation**	Financial problem	34	60.7
	Treated at peripheral hospitals	32	57.1
	Treated by traditional healers	31	55.4
	Lack of money for transport	31	55.4
	Self medication at home	12	21.4
	No reasons documented	12	21.4
**Site of the lesion**	Lower limb	24	42.9
	Head & neck	13	23.2
	Upper limb	12	21.4
	Trunk	4	7.1
	Genitalia	2	3.6
**Symptoms**	Increasing pain	52	92.9
	Foul smelling pus discharge	50	89.3
	Haemorrhage	36	64.3
	Increasing discharge	35	62.5
	Increasing tumor size	30	53.6
**Tumor type**	Exophylic	27	48.2
	Infiltrative	29	51.8
**Tumor size**	Less than 5 cm diameter	8	14.3
	5 cm or more diameter	48	85.7
**Metastasis at the time of diagnosis**	Lymph node metastasis	18	26.9
	Distant metastasis	15	32.1
	No evidence of metastasis	25	44.6

The lower limb was the most frequent site for Marjolin's ulcers accounting for 42.9% of patients. The majority of patients presented with increasing pain (92.9%) and foul smelling pus discharge (89.3%) and most of them (51.8%) had exophytic proliferative ulcers. The median tumor size at presentation was 8 cm (range 2 to 16 cm) and the vast majority of patients (85.7%) presented with large tumors of ≥ 2 cm in diameter. Most patients (44.6%) had no evidence of metastases. Lymph node metastasis at the time of diagnosis was recorded in 32.1% of cases. Distant metastasis accounted for 26.9% of cases and occurred mostly in the lungs, liver, bone and brain in 6 (40.0%), 5(33.3%), 2 (13.3%) and 2 (13.3%) patients respectively (Table 1).

### Investigations

A total of 70 radiological investigations were performed to rule out distant metastasis; of these, plain radiographs were the most frequent radiological investigation performed accounting for 64.3% of all the radiological investigations (Table 2). Table 3 shows histopathological findings of various Marjolin's ulcers. Squamous cell carcinoma was the most common histopathological type accounting for 91.1% of all Marjolin's ulcers. The majority of these tumors (Marjolin's ulcers) were moderate differentiation in 41.1%. The depth of dermal invasion and vertical tumor thickness is shown in Table 3.

**Table 2:** Radiological investigations (N = 70)

Radiological study	Frequency	Percentage
**Plain radiographs**	**45**	**64.3**
Long bone x-rays	28	62.2
Chest x-rays	18	40.0
Skull x-rays	12	26.7
**Abdominal ultrasound**	**16**	**22.9**
**Computed Tomography (CT) scan brain**	**9**	**12.9**

**Table 3:** Histopathological results (N = 56)

Variable	Response	Frequency	Percentage
**Histopathological type**	Squamous cell carcinoma	51	91.1
	Basal cell carcinoma	2	3.6
	Osteogenic sarcoma	1	1.8
	Fibrosarcoma	1	1.8
	Melanoma	1	1.8
**Tumor grade**	Well differentiated	16	28.6
	Moderate differentiated	23	41.1
	Poor differentiated	17	30.3
**Depth of dermal invasion**	Superficial to reticular dermis	4	7.1
	Reticular dermis or deep	36	64.3
	Not documented	16	28.6
**Vertical tumor thickness**	Less than 4 mm thick	9	16.1
	4 mm thick or more	35	62.5
	Not documented	12	21.4

### Treatment modalities

In the present study, fifty-two (92.9%) patients underwent surgical treatment. Of these, wide local excision was the most common surgical procedure performed in 80.8% of cases (Table 4).

**Table 4:** Type of surgical procedures performed (N = 52)

Type of surgical procedure	Frequency	Percentage
**Wide local excision**	**42**	**80.8**
With skin grafting	36	85.7
With flap	6	14.3
**Limb amputation**	**6**	**11.5**
Minor	2	33.3
Major	4	66.7
**Lymph node dissection**	**4**	**7.7**

### Clinical Outcome

Post-operative complications were recorded in 18 (32.1%) patients. Of these, surgical site infection was the most common post-operative complication accounting for 38.9% of cases (Table 5). The majority of patients, 51 (91.1%) required hospitalization for their treatment and the remaining 5(8.9%) were treated as outpatients. The length of hospital stay for in-patients ranged from 6 to 21 days (mean 7.9 ± 2.3 days). Four patients died in hospital giving a mortality rate of 7.1%. According to multivariate logistic regression analysis, stage and grade of the tumor, presence of metastases and presence of local recurrence were the main predictors of death (P < 0.001). Local recurrence was noted in 33.3% of cases who had surgical treatment. All cases of local recurrence of ulcers occurred from 6 months to 1 year (mean 8.3 ± 3.4 months) after definitive treatment. Tumor size, histological grade of the tumor, stage of the tumor and presence of metastasis at the time of diagnosis were the main predictors of local recurrence (P < 0.001).

**Table 5:** Postoperative complications (N = 18)

Postoperative complications	Frequency	Percentage
Surgical site infection	7	38.9
Local recurrence	6	33.3
Loss of skin grafting	2	11.1
Loss of flaps	1	5.5
Stump dehiscence	1	5.5
Re-amputation	1	5.5

## Discussion

Marjolin's ulcer is defined as a tumor arising from a chronic wound, scar or chronic inflammation [1,2]. Jean Nicholas Marjolin first described the malignant transformation of cutaneous scars in 1828 [1-3]. Since then, several reports of post-burn scar ulcers have been reported [7,8,11]. Various studies indicate that Marjolin's ulcers make up 1.2% of all skin cancers [7,17].

In this review, the mean age of patients was 38.2 years which is lower than the age reported in western countries [11,18,19]. Current studies in Africa have reported that the mean age of patients with Marjolin's ulcers is lowering and appears to be affecting younger patients over the years [12,20]. It also appears that the transition time is getting shorter [20]. Marjolin's ulcers in general, develop in younger patients amongst sub-Saharan patients than those reported from other regions [12]; therefore, patients presenting with chronic ulcers should be investigated during the initial evaluation for this possibility. Further research is needed in our region to explain this observation.

The two-fold increase in the number of males with Marjolin's ulcers compared to females in the present study is similar to what was reported in other studies [7,21,22]. Male preponderance in the present study may be due to their increased susceptibility to trauma which, if poorly managed, these lesions have been reported to undergo malignant transformation.

Marjolin's ulcer is a malignant change in a long-standing ulcer and/or scar tissue [22,23]. In agreement with other studies [4,7,11,14,16], the most frequent predisposing lesion of Marjolin's ulcers in the present study was post burn scars. Marjolin ulcers generally occur in regions of previous deep burn that healed slowly without skin grafting [10]. This observation is reflected in our study where only 12.0% of patients reported to have skin grafting for their previous causative lesions. Burn scar carcinoma has a propensity for the extremities, specifically to flexion creases of the extremities, where blood supply is decreased and vulnerability to trauma is increased [23]. Marjolin's ulcers have also been reported following other traumatic injuries, leg ulceration, chronic sinuses of osteomyelitis, pressure sores, and discoid lupus erythematosus [24].They have also been documented in the genitalia as a complication of Fournier's gangrene [13]. Interestingly, one patient in our study was a rare case of a 33 year-old patient who presented with an early appearance of Marjolin's ulcer developing in a penile human bite scar seven months after the initial injury.

The precise mechanism by which chronic ulcers (wounds) develop malignancy is not known and many theories have been postulated [9]. Early theories suggested that cellular mutations as a result of inflammatory related substances release by damaged, ischemic and nutritionally deficient tissues are responsible for neoplastic change [4]. Neuman *et al *[25] proposed that traumatic displacement of living epithelial tissue into dermis may cause a foreign body response and lead to a deranged regenerative process, resulting in carcinomatous change. More recently, a theory of immunologic isolation has been suggested, whereby lymphatic channel obliteration at the site of injury decreases the delivery of antigen or specifically stimulated small lymphocytes to the regional lymphocytes from that site. This renders the site 'immulogically unprivileged', allowing the development of antigenically foreign tumor cells to go unchecked. Such cells may initially arise by spontaneous mutation or develop under the influence of viral or chemical carcinogens. Tumor antigen recognition may then be delayed long enough for tumors to reach 'critical size', when immune mechanisms are no longer sufficient to prevent continued neoplastic progression [26]. It has also been suggested that patients with an inherent immune deficiency are at higher risk for developing malignant ulcers [27]. Some authors have also postulated that with chronic irritation and repeated damage of the ulcer, there is continuous mitotic activity as the epidermal cells attempt to resurface the open defect. This cycle of damage, irritation, and repair can lead to a malignant transformation [28]. More recent theories have included genetic postulations involving human leukocyte antigen (HLA) DR4 and mutations in the p53 and/or Fas genes [29-32].

Two variants of Marjolin's ulcer have been described; an acute form, in which the cancer occurs within one year of the injury and a chronic form in which malignant changes are more than one year from the date of injury. The chronic form is more frequent and malignancy tends to develop slowly, with an average latency period of 36 years [21,33]. The younger the patient at the time of injury, the longer the interval for malignant change, while the older the patient at the time of burning, the longer the lag period. The results of this study showed an average latency period of 11 years which is lower than the latency period reported in most western studies [9,22]. It has been observed that the latency period is inversely proportional to the patients' age. The reason for this phenomenon is unknown.

In keeping with other studies [4,18,21,24,34,35], the lower limb was the most frequent site for Marjolin's ulcers. The anatomical locations reported by Arons [36], Lawrence [37], and Novick [38] show that average distribution of Marjolin ulcers is 40% in the lower extremity, 30% in the head and face, 20% in the upper extremity, and 10% in the trunk area. The reason for this anatomical site predilection is not well understood.

Marjolin's ulcers are commonly mistaken for an infected ulceration occurring at the scar tissue sites and may often be overlooked. Changes such as the appearance of flat, non-healing ulcers enlarging in circumference with elevated and indurated borders, foul-smelling, painful with exudates and bloody drainage suggest a malignant transformation [39,40]. As in other studies [8,9,22,39,40], the majority of our patients in the present study presented with increasing pain, foul smelling pus discharge, increasing tumor size and most of them had exophytic proliferative ulcers. It is therefore recommended that all chronic ulcers should be thoroughly investigated at presentation, to avoid labeling malignancies 'chronic ulcers', leading to delay in appropriate treatment. All patients presenting with chronic ulcers should undergo multiple biopsies, to help confirm the neoplasmatic conversion and to avoid missing malignant ulcers.

Macroscopically, Marjolin's ulcers have been reported to exist in two forms which are of prognostic importance1) *Exophytic form *characterized by prolonged and relatively benign course and low probability of distant metastasis 2) *Infiltrative form *characterized by rapid formation of ulceration and worse prognosis and high probability of metastatic spread [22]. Infiltrative Marjolin's ulcers was the most common form in the present study accounting for 51.8% of cases and this may be responsible for the high rate of metastatic spread in our study.

Marjolin's ulcers have been reported to have an aggressive course and a much greater tendency to metastasize than other types of skin cancer, which makes early diagnosis imperative. Metastasis to the brain, liver, lung, kidney, and distant lymph nodes has been commonly reported [8,21,34,35,41]. In the present study, 32.1% of patients had lymph node metastasis at the time of diagnosis and 26.9% of cases had distant metastasis to the lungs, liver, bone and brain, the rate which is higher than that reported in other studies [34,35]. High lymph node and distant metastasis in our study is due to the fact that most patients in the present study present late when the disease is already in advanced stages. In developing countries like ours, especially in rural areas with poor living conditions most patients are already in advanced stages of disease at the time of diagnosis of Marjolin's ulcer, which has been proven both in the present study and in literature [42,43]. In the present study, more than 80% of patients presented late with large tumor > 2 cm in diameter. Late presentation is common in most developing countries as a result of poverty, ignorance, and poor referral systems in a relatively expensive health care system devoid of meaningful health insurance [12]. Financial problem and delayed referral to tertiary health care facility were the most common reasons for late presentation in the present study. Health education is highly needed to discourage patients with cutaneous ulcers from presenting late to hospital when the disease is in its advanced stage. Early adequate treatment of all ulcers and scars will certainly reduce the incidence of Marjolin's ulcer in our setting.

The commonest histopathological type of carcinoma arising from Marjolin's ulcer is squamous cell carcinoma, followed by basal cell carcinoma as the second commonest carcinoma [7,8,10,16,16,18,22,23]. This finding is in agreement with the present study in which more than 90% of Marjolin's ulcers were squamous cell carcinoma. Other reported neoplasms are malignant melanoma, osteogenic sarcoma, fibrosarcoma and liposarcoma [8,16].

The treatment of Marjolin's ulcers requires multidisciplinary approach. Treatment modalities of Marjolin's ulcers include wide local excision, block dissection of the regional nodes, amputation in advanced lesions of limbs, radiotherapy and chemotherapy given either as neo or adjuvant therapy [10]. Wide local excision (surgical margin of at least 2 cm), together with skin grafting primarily or primarily delayed, is usually considered appropriate in the treatment of Marjolin's ulcers [10,40]. Adequate surgical resection is most important to prevent local recurrence and a margin of 2-5 cm has been advocated [12,40]. Frozen sections have been reported to be used for intraoperative diagnosis and evaluation of surgical excision safety margins [17]. However, like in most developing countries, frozen sections are not performed in our centre partly because of few available pathologists and lack of facilities for performing frozen sections.

Amputation is indicated when wide local excision is not possible due to deep invasion, bone or joint involvement, infection, or hemorrhage, or when excision would cause major functional disability. Regional lymph node dissection is indicated when nodes are clinically palpable with an exception for malignant melanoma, where the sentinel lymph node biopsy should be performed regardless of the presence of enlarged lymph nodes [40]. Sentinel lymph node biopsy has been shown to give as high yield as 83% [40,44] and is recommended to detect occult nodal involvement. However, sentinel lymph node biopsy was not performed in the present study due to its unavailability in our centre. In agreement with other studies [10,40], wide local excision with either skin grafting or flap coverage, was the most frequent surgical procedure performed in the present study. Limb amputation and lymph node dissection were performed in 11.5% and 7.7% of cases. Radiotherapy and chemotherapy (in the form of four courses of (Methotrexate, Bleomycin and Cisplatinum) is indicated in patients with poor prognostic factors or distant metastasis [24,36]. In our study, patients who had inoperable tumor and those who had recurrences were referred to Oncological centre for possible palliative radiotherapy/chemotherapy.

Most series indicate that the incidence of recurrence is in the range of 20% to 50% [16,24]. Most recurrences are regional, but metastases to the brain, liver, lung, kidney, and distant lymph nodes have been reported [24]. In the present study, local recurrence was recorded in 33.3% of cases which is higher rate than that is reported by other authors [8,21,34,35,41]. High recurrence rate in our study is attributed to delayed presentation and diagnosis and this confirmed the highly metastatic potential of Marjolin's ulcer among skin malignancies.

Recurrence and fatality rates are higher due to the aggressive nature of this tumor [28,33].

Our overall mortality rate in the present study was 7.1% that is relatively lower than that reported in other studies [35,44]. In the present study, mortality rate was significantly high in patients with high stage and grade of the tumor and those who had metastases and local recurrences reflecting the aggressive nature of this tumor.

The prognosis in Marjolin's ulcers depends on various factors like age of the patient, size, grade and stage of the tumor, presence of metastases, the adequacy of surgery and presence of local recurrence. In the present study, stage and grade of the tumor, presence of metastases and presence of local recurrence were the main predictors of death. Appropriate Marjolin's ulcer patient prognostication is of paramount important in clinical decision making, especially the utilization of resources in poor income countries.

## Conclusion

Marjolin's ulcers are not uncommon in our setting and commonly occur in burn scars that were not skin grafted and were left to heal secondarily. They have a shorter latent period and commonly occurred in younger male patients. Most patients in our environment present late when the disease is already in advanced stages. Recurrence rate following surgical treatment was 33.3%. A high index of suspicion is required in the management of chronic non-healing ulcers that are recalcitrant to therapy and all suspected lesions should be biopsed. Early recognition and aggressive treatment of Marjolin's ulcers and close follow-up are urgently needed to improve outcomes in our environment. Health education is highly needed to discourage patients with cutaneous ulcers from presenting late to hospital when the disease is in its advanced stage.

## Competing interests

The authors declare that they have no competing interests.

## Authors' contributions

PLC contributed in study design, literature search, data analysis, manuscript writing, editing and submission of the manuscript. JBM, PR, ABC, GG and KJK participated in study design, data analysis, manuscript writing & editing. MDM participated in data analysis, manuscript writing & editing. NM and RS participated in data analysis and manuscript writing. JMG supervised the study and contributed in data analysis, manuscript writing & editing. All the authors read and approved the final manuscript.
